# Pomological and Molecular Characterization of Apple Cultivars in the German Fruit Genebank

**DOI:** 10.3390/plants13192699

**Published:** 2024-09-26

**Authors:** Lea Broschewitz, Stefanie Reim, Henryk Flachowsky, Monika Höfer

**Affiliations:** Julius Kühn Institute (JKI), Federal Research Centre for Cultivated Plants, Institute of Breeding Research on Fruit Crops, Pillnitzer Platz 3a, 01326 Dresden, Germany

**Keywords:** *Malus* × *domestica*, trueness-to-type, genetic diversity, genetic structure, parentage analysis

## Abstract

Traditional varieties are a valuable tool in modern apple breeding. However, the use of synonyms and missing source documentation hinder an effective identification and conservation of relevant cultivars. During several projects, the authenticity and diversity of the apple cultivar collection of the German Fruit Genebank (GFG) was evaluated extensively. The trueness-to-type of 7890 apple trees was assessed on a pomological and molecular level. Pomological evaluations were performed by at least two experienced experts to identify the original cultivar names. On the molecular level, a set of 17 SSR markers was used to determine a unique genetic profile for each apple cultivar. The pomological and molecular characterization was expressed in terms of a comprehensive trueness-to-type criterion and the results were previously published as a well-curated dataset. In this study, the published dataset was analyzed to evaluate the quality and diversity of the apple collection of the GFG and highlight new findings based on phylogenetic and parentage analysis. The dataset contains 1404 unique genetic profiles corresponding to unambiguous cultivar names. Of these 1404 cultivars, 74% were assessed as true-to-type. The collection of diploid apple cultivars showed a high degree of expected heterozygosity (*H_exp_* = 0.84). Genetic diversity in terms of year and location of origin was investigated with a STRUCTURE analysis. It was hypothesized that genetic diversity might decline overtime due to restrictive breeding programs. The results showed a shift dynamic between older and newer cultivars in one specific cluster, but no significant decrease in genetic diversity was observed in this study. Lastly, a parentage analysis was performed to check parental relationships based on historical research. Out of 128 parent–child trios, 110 trios resulted in significant relationships and reconfirmed the information from the literature. In some cases, the information from the literature was disproven. This analysis also allowed for readjusting the trueness-to-type criteria for previously undetermined cultivars. Overall, the importance of authenticity evaluations for gene bank cultivars was highlighted. Furthermore, the direct use of the dataset was shown by relevant investigations on the genetic diversity and structure of the apple cultivar collections of the GFG.

## 1. Introduction

Worldwide, over 90 million tons of apples (*Malus* × *domestica*) are produced yearly, making them the third most important fruit crop after banana and watermelon [1]. While large quantities of apples are farmed, commercial cultivation is limited to only a few varieties. In Germany, about 30 cultivars make up the main part of commercial apple farming [2,3]. Furthermore, apples are the most consumed fruit crop, led by popular cultivars such as ‘Elstar’, ‘Jonagold’, ‘Gala’ and ‘Braeburn’ [4,5]. The diversity of apple cultivars far exceeds this number. Back in the middle of the 19th century, around 2000 apple cultivars could be found in Germany [3]. While many cultivars still exist, the number of used cultivars has declined drastically. This phenomenon is rooted in the practice of commercial farming and food scarcity after the Second World War, which promoted the cultivation of selected high-yielding varieties [3].

In more recent times, the fruit industry has been challenged by the demand for organic and sustainable production while being confronted with the rising problems of climate change [6]. In breeding programs, this calls for novel, robust and disease-resistant cultivars [7]. Systematic breeding in Germany started around the 1920s and, subsequently, the interest in collecting cultivars and wild species of apple grew [8]. On the other hand, intensive selection breeding contributes to the loss of genetic diversity, which is why conserving genetic resources is essential for the diversification of possible breeding material. Therefore, genebank establishment and management play a crucial role in the development of future crops [9,10,11]. Valuable genetic resources for such breeding programs are provided by landraces, older apple varieties and wild crop relatives, which currently can be found in meadow orchards and dedicated collections.

One such collection is the German Fruit Genebank (GFG, https://www.deutsche-genbank-obst.de/), which manages the preservation of diverse cultivars of native fruit species, including apple. The GFG is a national decentralized network which aims for efficient long-term conservation while also making fruit genetic resources available for breeding and research [12]. The relevant cultivars for the GFG are “(1) German cultivars including new German selections, (2) cultivars with a social cultural, local or historical relation to Germany, and (3) cultivars with important pomological traits especially for breeders” [12]. The apple network is a special network dealing solely with *Malus* ssp. cultivars. The collections of the GFG partners of the apple network share various sources lacking a proven cultivar identity. Additionally, synonyms arise and spread while the original cultivar name is lost. Consequently, verifying the cultivar authenticity of the GFG apple collection becomes essential. This authenticity was assessed through a combination of detailed pomological and molecular characterizations in previous projects, with the resulting data published at https://www.openagrar.de/receive/openagrar_mods_00092714 [13]. This extensive two-step approach of reliably determining cultivar identity while supplementing it with molecular data has not been reported in the scientific literature so far. The focus often lies on genetic authenticity and comparability between accessions of historical and established collections, without reflection on the available literature and expertise on cultivars to reconfirm their identity [5,14,15]. Pomological characterization is essential to provide the correct cultivar description to the corresponding molecularly analyzed genotype. For molecular analysis, simple sequence repeat (SSR) markers are one of the oldest yet reliable methods of genotyping and are used for crop cultivar identification [16,17,18,19]. By using the reference set of SSR markers for apple cultivars, comparability to international datasets is possible [20,21,22].

Through the efforts of two-step cultivar authentication via pomological and molecular characterization, a well-curated dataset of 1404 apple genotypes was compiled, indicating the reliability status of cultivar identification in combination with a respective SSR marker genotype [13]. The work presented in this study is based on this dataset (https://www.openagrar.de/receive/openagrar_mods_00092714). The aims of this study were (I) to determine the trueness-to-type of apple cultivars in the GFG’s apple collection, (II) to assess the genetic diversity of diploid cultivars, (III) to examine the genetic structure of apple cultivars in the context of their age and origin and (IV) to perform a parentage analysis to provide insight on previously assumed parent–child relationships. While attaining these goals, the importance of the historical literature and cooperation with knowledgeable pomologists is highlighted and discussed.

## 2. Results

### 2.1. True-to-Type Apple Cultivars across GFG Collection

**Figure 1:**
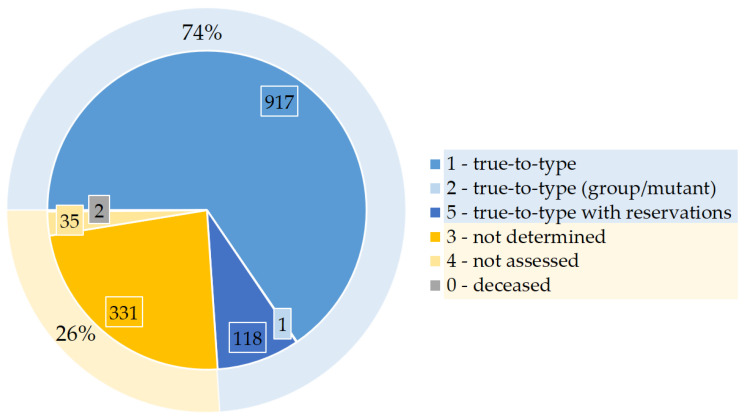
Representation of 1404 apple genotypes corresponding to cultivars in the German Fruit Genebank (GFG) sectioned by authenticity of cultivar identification (trueness-to-type) given in percentage and count.

Based on the published dataset (https://www.openagrar.de/receive/openagrar_mods_00092714), the GFG apple collection carries a total of 1404 apple genotypes (Supplemental Table S1) [13]. The authenticity of the cultivars is expressed in a specified scoring system (Figure 1; see Section 5.1 for a detailed explanation of trueness-to-type criterion). An overview of the 1404 unique cultivars reveals that 74% are known and true-to-type (Figure 1). This 74% includes 917 true-to-type cultivars, 1 mutation genotype (‘York Imperial Colora Red York’) and 118 cultivars that are considered true-to-type but with reservation.

The remaining 26% translates to 368 cultivars that have not been confidently authenticated yet. In 331 cases, the assumed cultivar was disproven or the cultivar name was unknown to the experts and the true cultivar name could not be determined so far. Another 35 cases could not be assessed because the apple trees did not develop fruit during the evaluation period. The trees of the remaining two cultivars died during the project duration and could not be assessed by the experts.

### 2.2. Dataset Assessment and Power of SSR Markers

**Figure 2:**
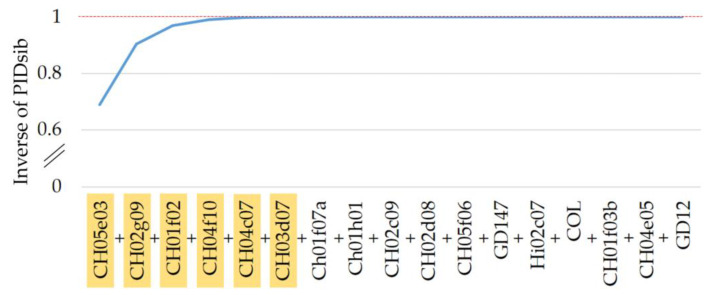
Combined effect of discrimination power of 17 SSR markers represented by average non-exclusion probability of identity of two unrelated individuals (PID) and average non-exclusion probability of identity of two siblings (PIDsib). The markers required for near 100% discrimination of the apple genotypes are marked in yellow.

Out of 1404 cultivars, 1085 were determined to be diploid. The inspected dataset of 1085 genotypes has a high genetic diversity, expressed by a mean expected heterozygosity of *H_exp_* = 0.84 and a mean number of alleles per locus of 22.06. The numbers of alleles ranged from *N_a_* = 14 for CH05f06 and CH01f03b to *N_a_* = 38 for CH05e03 (Table 1). The mean number of effective alleles was *N_e_* = 6.56, where the lowest *N_e_* = 3.36 was calculated for GD12 and the highest *N_e_* = 9.01 for CH05e03 and CH02g09. The expected heterozygosity across different markers varied from *H_exp_* = 0.7 for GD12 to *H_exp_* = 0.89 for CH05e03 and CH02g09. The SSR markers used in this study showed a high discriminatory power, as indicated by the polymorphic information content (mean PIC = 0.82). The highest PIC was given for SSR markers CH05e03 and CH02g09 (PIC = 0.88), while GD12 (PIC = 0.68) had the lowest value. The high discrimination power is also supported by the low values for the average non-exclusion probability of identity of two unrelated individuals (PID) and average non-exclusion probability of identity of two siblings (PIDsib) presented in Table 1. Here, the mean PID and PIDsib yielded values of 0.05 and 0.34, respectively. The cumulative effect of the combination of SSR markers in distinguishing genotypes is depicted in Figure 2. Due to the context of the dataset, it is expected that the apple cultivars are closely related and PIDsib is the parameter of choice. When combining the markers in ascending order of PIDsib, it can be seen that a distinction for almost 100% of the genotypes is reached with six markers. Specifically, these important markers are CH05e03, CH02g09, CH01f02, CH04f10, CH04c07 and CH03d07. The necessity of at least six markers to reliably separate the given 1085 genotypes was also expressed by a genotype accumulation curve (Supplementary Figure S1).

### 2.3. Genetic Structure of Apple Cultivars in Context of Age and Origin

To investigate the possible decrease in genetic diversity in modern apple cultivars, the genetic diversity and structure of 569 varieties were analyzed with age data from the database of the GFG, which is mainly based on (historical) data from the literature [23,24]. These apple cultivars were diploid and could be categorized into four different age groups based on the time of emergence, first mention or documented breeding year (Table 2; see Section 5.3 for overview of groups; Supplementary Table S1). The age groups had similar numbers of members, ranging from 137 to 156. The expected heterozygosity *H_exp_* of the age groups ranged from 0.81 to 0.84, showing no significant change across groups (Table 2). The Nei genetic identity between the age groups was also very high (>0.89), indicating a high variability of the overall dataset but not between the individual groups. The average number of alleles *Na* decreases slightly over time, which is in line with the subtle decrease in *H_exp_*.

**Figure 3:**
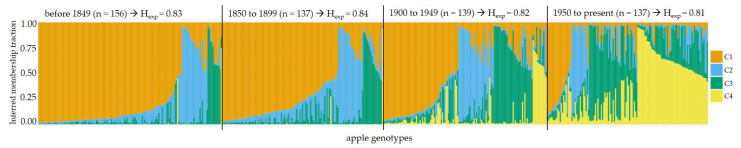
Genetic structure of 569 apple genotypes based on STRUCTURE analysis of 17 SSR markers at predefined K = 4. Each genotype is represented by a vertical bar that is partitioned in color according to the inferred membership fraction to clusters 1 to 4 (C1 to C4). The data are sorted by Q-value per age group. The expected heterozygosity (*H_exp_*) per age group is given. Age groups refer to the respective age range of the cultivars.

In the genetic structure analysis, four clusters (C1–C4) were formed using the age group information (Figure 3). The distribution of apple cultivars with comparable inferred membership fractions is very similar between the “before 1849” and “1850 to 1899” age groups (Figure 3). It can be observed that more than 70% of individuals of the “before 1849” and “1850 to 1899” age groups are sorted to group C1 (Table 3). All other cluster proportions for the members of these two age groups range between 15.3% and 0.6%. This trend of predominant classification is not as apparent for groups “1900 to 1949” and “1950 to present”. Although 46% of “1900 to 1949”cultivars are also grouped with C1, proportions of more than 20% are also grouped with C2 and C3. For group “1900 to 1949”, a proportion of C4 (8.6%) can be observed, which was very small in groups “before 1849” and “1850 to 1899” (<0.8%). The members of “1950 to present” are predominantly grouped in C4 (43.8%) and C3 (29.9%), which separates this group from the others. This emerging shift of C4 in groups “1900 to 1949” and “1950 to present” is also well visualized in Figure 3. C4 is highly under-represented in groups “before 1849” and “1850 to 1899”, with only one cultivar each: ‘Jonathan’ (MUNQ 57) and ‘Golden Delicious’ (MUNQ 65), respectively. In the “1900 to 1949” age group, the proportion of C4 increases, while that of C1 decreases. Then, for “1950 to present”, there are more members grouped with C4 than with C1. The proportion of the clusters, and therefore genetic structure, is clearly dynamic when put in the context of cultivar age.

**Figure 4:**
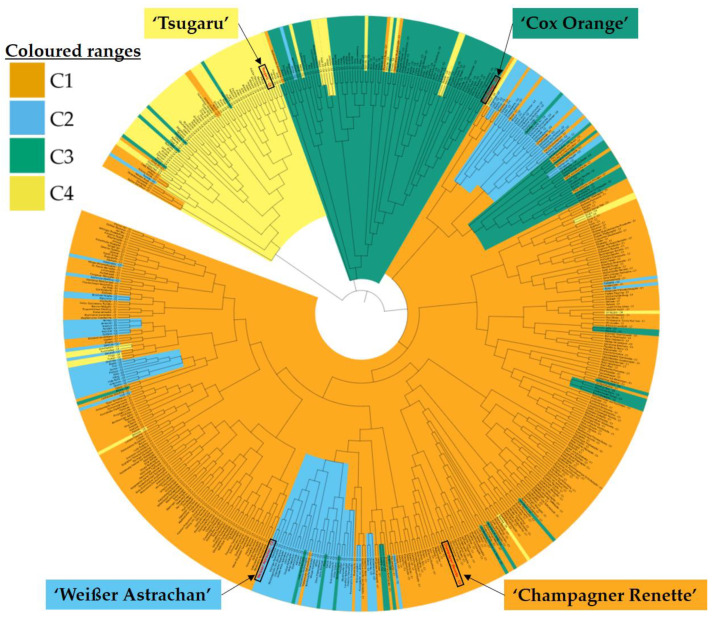
Phylogenetic tree of the genetic structure analysis results. Genetic data of 17 SSR markers of 569 apple cultivars were used for tree construction using the neighbor-joining method. Cultivars are marked according to their respective dominant cluster (C1 to C4; Clusters 1 to 4) based on the inferred membership fraction. In each cluster, the cultivar with the highest inferred membership fraction is marked with red font color and additional labels. A high-resolution image is provided in Supplementary Figure S2.

The uneven distribution of cultivars across the created clusters can be observed in Figure 4 (see Supplementary Figure S2 for higher resolution). C1 was composed of 306 out of the total 569 cultivars (Supplementary Text File S1). In the phylogenetic tree, C4 branched off distinctly. Most members of C3 can be found in a separate branch, with the exception of one subgroup. The rest of the phylogenetic tree is dominated by C1. Members of C2 build sub-branches that underlie C1. The apple cultivars with the highest proportions of C1, C2, C3 and C4 are ‘Champagner Renette’ (MUNQ 91), ‘Weißer Astrachan’ (MUNQ 24), ‘Cox Orange’ (MUNQ 163) and ‘Tsugaru’ (MUNQ 1339), respectively (marked in Figure 4). For C3, the nine consecutive cultivars with the highest cluster membership fraction are all descendent of ‘Cox Orange’. Similarly, the top eight consecutive C4 cultivars are directly related to ‘Golden Delicious’, except for ‘Aiwanija’ (MUNQ 4745), as there is no information on its parentage. Such a strong correlation cannot be observed for C1 and C2. For none of the top ten cultivars in C1 is parentage information given. The only common feature is that five out of the ten are described as “Renette”, a historic class of apples based on a distinct flavor. Here, ‘Französische Edelrenette’ (MUNQ 278) represents one of the oldest cultivars of the dataset, dating back to 1510. Parentage information was also limited for C2 but, when given, indicated a connection to ‘Weißer Astrachan’, ‘Weißer Klarapfel’ (MUNQ 25) and ‘McIntosh’ (MUNQ 508).

Another interesting dynamic can be observed when putting the genetic structure analysis data in the context of geographical origin. Therefore, apple cultivars were sorted into groups “Germany”, “Europe” and “Other”, indicating the place of discovery or breeding. Here, a dynamic was discovered in group C3 (Supplementary Figure S3). While C3 is represented by more than forty-five cultivars in the “Germany” and “Europe” groups, there are only five individuals in the “Other” group. These cultivars are ‘Bismarckapfel’ (MUNQ 3), ‘Bonza’ (MUNQ 5318), ‘Demokrat’ (MUNQ 112), ‘Sansa’ (MUNQ 1224) and ‘Rebekka’ (MUNQ 7991). In addition, C1 is under-represented in the “Other” group (Supplementary Table S2). Contrary to the decrease in C3 and C1, the proportions of C2 and C4 seem stable across the origin groups considering the differing sample sizes. A Q-plot sorted in accordance with the origin groups and a visualization of the described dynamic can be found in Supplementary Figure S3.

### 2.4. Parent-Child-Relationships between Apple Cultivars

A pressing issue for breeders is documenting the lineage of apple cultivars to avoid genetic bottlenecks. The documentation of apple lineage information can be revised based on molecular data, i.e., by parentage analysis. In this study, out of 1085 diploid genotypes, 253 cultivars had information on their parentage from a wide range of references in the literature [23]. For 128 genotypes, both parents were given, while for 100 and 25 genotypes, only the mother and father were given, respectively. Adding the offspring and paternal cultivars together, a total of 315 genotypes were used in the analysis (Supplementary Table S1).

The parentage analysis confirmed the full parentage for 110 cultivars out of the 128 cultivars for which information was provided (Table 4). For all 110 cultivars, the confidence level was at least 95%. Out of the remaining 18 cultivars, only the mother was confirmed for 12, meaning the paternal genotype was disproven (Table 5, Supplementary Table S3). For three cultivars, ‘Dukat’, ‘Jongrimes’ and ‘Kurzcox’, only the respective fathers were statistically supported, while the respective mothers, ‘Golden Delicious’, ‘Jonathan’ and ‘Königlicher Kurzstiel’, were rejected. Lastly, for ‘Collina’, ‘Erwin Junge’ and ‘Murasaki’, both suggested parents were not approved.

For 100 cases for which the maternal cultivar was given, this could be confirmed for 82 cases (Table 5, Supplementary Table S3). The remaining 18 cultivars were ‘Alexis’, ‘Arkcharm’, ‘Borsdorfer Kitajka’, ‘Coop 39’, ‘Cornwalliser Nelkenapfel’, ‘Crimson Beauty’, ‘Deliclious’, ‘Dietzer Wintergoldrenette’, ‘Korei’, ‘Lombarts Kalvill’, ‘Martini’, ‘Resista’, ‘Safranpepping’, ‘Schöner aus Miltenberg’, ‘Signe Tillisch’, ‘Sunrise’, ‘Uhlhorns Augustkalvill’ and ‘Werdersche Wachsrenette’ and their respective mothers were rejected statistically.

Lastly, for 18 out of 25 cultivars, the paternal cultivar was proven based on the molecular data (Table 5, Supplementary Table S3). The respective suggested fathers for seven cultivars, ‘Edward VII’, ‘Gartenmeister Simon’, ‘Howgate Wonder’, ‘Lane’s Prince Albert’, ‘Oetwiler Renette’, ‘Pomfital 1’ and ‘Schöner aus Elmpt’, were not statistically supported.

Parentage analyses are also valuable in the context of cultivar identification and confirmation. Out of the 110 cultivars with statistically confirmed full parentage, there were 2 cultivars with uncertain cultivar identity, namely ‘Holiday’ and ‘Shinsei’. The parentages of ‘Holiday’ (‘Macoun’ × ‘Jonathan’) and ‘Shinsei’ (‘Golden Delicious’ × ‘Early McIntosh’) referenced in the literature were proven in this study [25]. While the identity of ‘Holiday’ and ‘Shinsei’ was uncertain, the cultivar identity of the respective parents was validated. Therefore, the cultivar identity for ‘Holiday’ and ‘Shinsei’ can also be adjusted to a higher level. Similarly, the cultivar ‘Lady Williams’ could be classified as true-to-type after this analysis. In the literature, ‘Lady Williams’, evaluated as uncertain, is a mother to ‘Cripps Pink’ and ‘Cripps Red’, which are both true-to-type cultivars in the GFG collection [25]. The direct relationship in supported by the molecular data, and ‘Lady Williams’ could also be classified as true-to-type.

## 3. Discussion

### 3.1. The Importance of Cultivar Identification and the Cultivar Context

The main goal of the GFG is the conservation of genetic resources on native fruit species. Conservation can only be effective if the efforts and resources are targeting the appropriate cultivars. Therefore, an evaluation system for trueness-to-type was set up to ensure a comprehensive alignment between the plant material and the assigned cultivar name. To achieve this, several projects on different fruit species, e.g., sweet cherry [26], have already been completed, or are being conducted, e.g., on pear, plum or strawberry (JKI, unpublished). In this study, the published dataset of apple cultivars is presented and discussed [13]. A total of 74% of 1404 apple cultivars were deemed true-to-type (Figure 1). This adds up to 1036 apple cultivars, which are valuable genetic resources for the GFG.

For reliable validation, it was necessary to combine the pomological and molecular approaches. While the pomological approach is very consuming in terms of time and resources, it is essential for the correct link between the plant or molecular sample and the cultivar name. In addition, pomological assessment is still necessary for identifying mutations (sports) that cannot be distinguished by SSR markers. After establishing a reliable database for apple cultivars, including the genotype information, future cultivar identification can now be simplified by using only molecular data. If a cultivar is registered in the GFG with the respective genetic profile, plant material can be molecularly tested with the presented methods and aligned with the database to infer cultivar identity. Therefore, such a curated database becomes extremely relevant as the number of pomological experts is declining and molecular identification is becoming cheaper, as well as less time consuming. This information is especially relevant in the context of identity-by-descent analysis, which breeders use to infer the inheritance of quantitative trait loci and individual genes [27]. In breeding, such analyses rely heavily on correct knowledge about parentage and origin to predict the passing of traits from one generation to the next.

Also, expressing the authenticity of the cultivar by comprehensive trueness-to-type criteria is a unique feature of the GFG. During the process of apple cultivar identification, it became apparent that the assumed identity was not always true. Other international database providers and initiatives such as the ECPGR or INRAE (National Research Institute for Agriculture, Food and the Environment, Paris, France) also struggle to infer the true cultivar identity. The assignment of the internationally recognized MUNQ (Malus UNiQue genotype code) allows for the comparison of international genotype data through the harmonized use of microsatellite and single-nucleotide polymorphism markers [14,21,22]. Here, the cultivar names differ greatly between accessions of collections but could indicate the cultivar identity after extensive curation. Such alignments with larger databases are necessary to possibly identify cultivars that pomological experts could not name. Therefore, unifying existing data under a preferred cultivar name across the GFG, recognizing synonym names and conducting a reliability evaluation are essential achievements.

These achievements are also highlighted when looking at molecular data without the necessary context. For example, a parentage analysis purely based on genotype data can reveal direct relationships but without the historical context: it is not clear which individual is the progeny and which the ancestor. This assumption can only be made with the context information of, e.g., the cultivar name. The cultivar name is directly linked to many references in the historical literature and to information on cultivar origin, emergence, phenotype, etc. Eventually, coherent and logical conclusions can only be drawn when the used molecular data are correctly annotated. Hence, the GFG conducts essential work for future scientists and breeders by generating and curating a reliable database of native fruit species.

### 3.2. Genetic Diversity and Structure

The apple collection of the GFG was investigated for genetic diversity and structure to characterize its genetic resources. Each chosen marker displayed a high power of discrimination based on the polymorphic information content (Table 1). This makes the marker set suggested by the ECPGR (https://www.ecpgr.org/) suitable for the analysis of apple cultivars. The obtained dataset of apple cultivars was used to investigate their genetic diversity and structure. The investigation was limited to diploid genotypes based on the restrictions imposed by the used analysis tools (Supplementary Table S1). The apple genotype data showed a high genetic diversity (*H_exp_* = 0.84), and the markers used showed a high power of discrimination between the individual genotypes (Figure 2). The investigated parameters of the diploid dataset are comparable to other similar studies [28,29,30,31].

Breeders and pomologists are highly concerned with the possible decrease in genetic diversity in modern apple cultivars [32]. Fruit crop improvement took off in the 19th century, and apple breeding started to become commercialized in the early 20th century [33]. Yet the development of new apple cultivars is stunted by the common selection and propagation of mutated clones or sports [34]. These clones have a high chance of successful commercialization, especially when the mutation arises from an already popular cultivar. Furthermore, breeding and selection are often restricted to prominent cultivars such as ‘Golden Delicious’ or ‘McIntosh’, driven by customer preferences, which may contribute to a bottleneck in the germplasm. Therefore, a subset of diploid apple cultivars with known emergence or breeding age was compiled and analyzed for changes in the genetic structure over time (Supplementary Table S1).

The results showed that genetic diversity only decreased slightly over time, based on *H_exp_* = 0.83 for cultivars “before 1849” and *H_exp_* = 0.81 for “1950 to present”, and the respective allelic diversity was reduced from *N_a_* = 15.65 to *N_a_* = 12.24 (Table 2, Figure 3). Apple cultivars showed very little variation across the different times, expressed by the high Nei genetic identity (Table 2). Interestingly, two identified clusters (C3 and C4) expressed a dynamic shift with higher proportions in more recent times (cultivars after 1900; Table 3). C3 and C4 are cultivar-specific and carry cultivars that are closely related to ‘Cox Orange’ and ‘Golden Delicious’, respectively. This was also reflected in the phylogenetic tree, where most cultivars of these clusters clearly belonged to established branches (Figure 4). Previous studies, e.g., Migicovsky et al. [34], Hokanson et al. [35] and Larsen et al. [36], already highlighted the prevalent genetic background of ‘Golden Delicious’ and ‘Cox Orange’ in modern cultivars, which is also reflected here. Incidentally, for C1 and C2, ‘Reinette Franche’ (=‘Französische Edelrenette’) and ‘White Astrachan’ (=‘Weißer Astrachan‘) were pointed out by Muranty et al. as cultivars involved in many parent offspring relationships in their dataset [14]. Also, Luby et al. inferred that “Reinette Franche’ was one of the main progenitor cultivars of not only European but also North American apple germplasms [37]. These results indicate that the formed clusters are based on progenitor cultivars, which change throughout time. Taking this into account, the assumption can be made that in the future, there will be changes again, but this does not mean a significant loss of genetic diversity. Currently, the cultivar ‘Honeycrisp’ is highly involved in breeding programs, which might be reflected in the genetic structure going forward [38].

This analysis showed that the underlying genetic structure is more dependent on the direct genetic origin, i.e., familial relations. This indicates that the genetic structure of apple cultivars is impacted by targeted breeding over time, and this could become more apparent as time goes on. Also, the genetic diversity of apple collections does not seem to be threatened, but, clearly, the diversity of commercialized apple cultivars at large is concerning. Nevertheless, this issue is due to factors such as consumer favoritism and growing conditions. Possibly, more cultivar-specific clusters could be derived by sub-clustering C1 and C2 and revealing more founder cultivars.

Additionally, the individual members of these clusters could be investigated in detail. For example, in C4, there is the ‘Aiwanija’ cultivar, for which few references in the literature and no parentage information are available. Since it clusters together with ‘Golden Delicious’ in cluster C4, a relationship can be inferred. In fact, the molecular data show that ‘Aiwanija’ is a direct relative of ‘Golden Delicious’ and, based on the respective crossing ages of 1957 and 1890, ‘Aiwanija’ is the progeny of ‘Golden Delicious’. So, genetic structure analysis can also be used to identify relationships between cultivars and aid in performing parentage analyses where—e.g., in Cervus—parent–child pairs should be initially suggested to simplify the analysis.

While the analysis of genetic structure was conducted by integrating the age information of the cultivars, the resulting data were also plotted to investigate a possible connection to the geographical origin (Supplementary Figure S3). It was hypothesized that geographic origin might also influence genetic structure, considering that the use of prominent cultivars might have been preferred in Europe but not outside of it [32,33]. Based on the highly variable abundance of cultivars from different regions, the dataset was roughly divided into German, European and other cultivars (see Section 5.3). The cluster proportion between “Germany and “Europe” was very similar (Supplementary Figure S3 and Table S2). Conversely to the findings of Urrestarazu et al. [29], clusters could not be directly associated with a specific geographic origin, which might be due to biased groups. In our study, mainly German cultivars were investigated (47% German, 36% Europe, 17% Other), whereas in the study of Urrestarazu et al. [29], genotypes from 16 different countries within Europe were investigated. Yet C3 showed interesting behavior. More than forty cultivars of C3 were present in “Germany” and “Europe” but only five in “Other”. This indicates that ‘Cox Orange’ and its relatives, as representatives for C3, are fairly common in Europe but not as far spread outside of it. In terms of the scion exchange of cultivars, the assumption can be made that cultivars with foreign ‘Golden Delicious’ background, i.e., C4, which are steadily present across origin groups, are widely used in Europe. Finally, this circles back to the descriptions of unilateral distribution and utilization of apple cultivars between Europe and America by Watkins [32] and Janick [33].

To summarize, the analysis of the genetic structure of apple cultivars over time indicates that the loss of genetic diversity so far is not as grave as expected. Nevertheless, development is greatly steered by breeding and breeders’ choices for individual crops. It is therefore wise to invest in genetic resources, and this highlights the important conservation work that is being carried out by gene banks such as the GFG. Additionally, it was shown that this analysis can be used to study and infer potential direct relationships between cultivars, which is especially relevant for cultivars that are not described in the literature. Lastly, origin was discussed as a factor in genetic structure. Unilateral distribution of ‘Cox Orange’-related cultivars was observed between European and non-European origins.

### 3.3. Assumed Parentages Confirmed for Most Apple Cultivars

The parentage analysis performed in this study confirmed the assumed parental cultivars for 110 out of the 128 cultivars for which both parents were given (Table 5). These findings are comparable to parentage analyses in other studies, such as Lassois et al. [28], Baric et al. [30] and Larsen et al. [36]. For cultivars such as ‘Alkmene’ (‘Geheimrat Dr. Oldenburg’ × ‘Cox Orange’), ‘Rubinola’ (‘Coop 2’ × ‘Rubin’) and ‘Auralia’ (‘Cox Orange’ × ‘Schöner aus Nordhausen’), parentage was proven based on molecular data [28,30]. In other cases, the current study can still be expanded. For example, for ‘Collina’, the suggested parents (‘Coop 4’ × ‘Elstar’) were rejected in this study, and Larsen et al. [36] identified ‘Santana’ as one possible parent, which was not tested here. The comparability of these studies is often impaired by the extensive use of synonyms, especially when dealing with international projects, given that cultivar names vary based on the language [26,39]. A meta-analysis of the performed parentage analyses could reveal interesting results, as it could also include both SSR marker studies and SNP marker studies and would eventually lead to more complete heritage descriptions. In the dataset used in this study, only the lead cultivar name was used, and we tried to adjust the parental information accordingly. Still, there are cultivars whose synonyms are widely used and overlap with other cultivars or their synonyms. This is also not always back-traceable to references found in the historical literature.

Another difficulty is that the apple accessions used in studies are often not evaluated for their trueness-to-type, and the results can be misleading and misinterpreted based on wrong cultivar names. Now, in the present study, it is reasonable to adjust the authenticity evaluation after the parentage analysis, as the original trueness-to-type evaluation was very elaborate. As presented in the results, cultivars ‘Shinsei’ and ‘Holiday’ can now be authenticated as true-to-type based on their true-to-type parents and successful parentage analysis (Supplementary Table S3). However, this procedure should not be used vice versa. One cannot conclude that the disproven parentages automatically mean that the assumed cultivars are wrong. References in the literature are not always reliable or precise in their description. For very old cultivars, such information can even be completely lost. This is where molecular confirmation is helpful but pomological assessment still remains essential. Apple is a monoecious, open pollinator, which complicates even the results of planned breeding programs. In the literature, as observed, the mother cultivar from which the descendent fruit was collected is predominantly known. The father cultivar can only be known if trees were hand-pollinated and protected afterwards. Here, an array of human errors can happen, from misidentifying the father cultivar to switching up the pollen [26,39,40,41]. Therefore, the direct interpretation of parentage analyses should be handled with caution. The parentage that was confirmed here is reliable since a well-curated database was used, yet we advise caution in automatically ruling out or re-identifying cultivars whose parentage was not proven.

## 4. Conclusions

The GFG fulfills an integral role in protecting valuable genetic resources of fruit crop species in Germany. Here, the apple cultivar collection of the apple network was explored in detail. The apple collection displayed a very high quality based on the 1404 described apple cultivars, of which 74% were described as true-to-type. The genetic diversity of apple cultivars was investigated, and it was shown that the loss of genetic diversity, which is often brought up by pomological experts, is not clearly traceable. Rather, genetic diversity shifts over time based on individual, dominant cultivars that build the genetic background. Additionally, the results of parentage analysis demonstrated that, on the one hand, molecular data can be used to reaffirm suggested parent–child relationships. On the other hand, information from the literature was also disproven, indicating the difficulty of the correct documentation of progeny for breeders. Still, this work also emphasized the importance of references in the historical literature and of pomological experts for providing essential context. Without such context, no meaningful conclusion will be drawn. This study wants to highlight the two-step approach to ensure cultivar identity, which does not only rely on molecular data but also incorporates pomological characterization. The presented inventory work evaluating the authenticity of apple cultivars was a key long-term goal of the GFG for ensuring the quality of the apple collection, as well as a proper allocation of means for the conservation of plant genetic resources. Finally, the use of aligned MUNQs provides necessary information for international comparability.

## 5. Materials and Methods

The work presented in this study relies heavily on a previously published dataset (https://www.openagrar.de/receive/openagrar_mods_00092714), as well as on information from the literature provided by the GFG [13,23]. A short abstract of the creational process of this dataset is described below.

On behalf of the German Federal Ministry of Food and Agriculture, five projects were conducted in two blocks from 2009 to 2014 and from 2017 to 2021 to determine the trueness-to-type of cultivars in the apple network of the GFG (funding codes: 2809BE006, 2809BE010, 2813BE001 2816BE007 and 2816BE008) [24,42]. In short, 7890 apple trees at eight locations across Germany were assessed pomologically by at least two experts of the German Pomological Association (Pomologen-Verein e.V., Hamburg, Germany) to infer the correct cultivar name. This procedure relied on the expertise of the pomologists, who used general fruit characteristics to identify the cultivars with respect to experience and references in the literature [24,43]. The pomological evaluation was supplemented by a molecular analysis performed by Microsynth ecogenics GmbH (Balgach, Switzerland). Seventeen SSR markers recommended by the Malus/Pyrus Working group of the European Cooperative Programme for Plant Genetic Resources (ECPGR; http://www.ecpgr.cgiar.org/) were used for genetic fingerprinting. Based on the set of SSR markers, internationally recognized MUNQ codes were aligned [21,22]. For each tree, there were pomological and molecular data, and they were compiled, resulting in a distinct genetic profile and trueness-to-type criterion per apple cultivar. After extensive data curation and data processing, a dataset of 1404 apple genotypes was compiled [13]. There is a distinction between the number of genotypes and the number of cultivars, as mutations or sports are only separable pomologically but not by SSR markers. Therefore, one genotype can represent several cultivars. Throughout this study, the terms genotype and cultivar are used interchangeably, and these instances are well reported in Supplementary Table S1. A data descriptor with further specification on this dataset was developed and will soon be published [13,44]. An overview of the necessary background information of the cultivars and their involvement in the individual analysis steps of this study can be found in Supplementary Table S1.

### 5.1. Trueness-to-Type Criterion

The trueness-to-type criterion was compiled based on the pomological and molecular results and was assessed on a scale from “0” to “5” (Table 6). On the one hand, cultivars were judged to be true-to-type if they scored a “1”, “2” and “5”. For criterion “1”, there is no doubt about the cultivar’s identity. Criterion “2” is as confident as “1” but is used for mutation cultivars. For example, true-to-type ‘Alkmene’ receives criterion “1”. Its derived red mutation ‘Rote Alkmene’ is also true-to-type but is assigned a “2”. Most of the mutation cultivars in the dataset are masked by the originating cultivar, as the genetic profile is non-distinguishable: for example, only ‘Alkmene’ is present, but not ‘Rote Alkmene’, as it is part of the same molecular group. When pomologists assigned a “5”, the cultivar was deemed true-to-type but without reservation. This might be, for example, due to contradicting references in the literature.

On the other hand, cultivars evaluated as “0”, “3” or “4” were deemed not true-to-type for the moment. Cultivars with “0” have unique cultivar names but the trees died during the projects; however, the cultivar could still be within the GFG collection, e.g., located at a different partner, and therefore the cultivar was not removed. Undetermined cultivars (“3”) might still be true-to-type but our pomological assessment could not classify them as such because of, e.g., missing (historical) references in the literature and missing information on newly bred, local or foreign cultivars. Evaluation with a “4” indicates that the cultivar could not be assessed pomologically due to no or unsuitable fruit development. Therefore, while all these three evaluation scores represent cultivars that are not true-to-type for now, these criteria can be adjusted with a new (pomological) assessment.

### 5.2. Probability of Identity Calculation and Diversity Parameters

Out of the 1404 cultivars, 1085 were determined to be diploid based on flow cytometry studies (data unpublished, JKI Dresden-Pillnitz) and showed only two alleles after SSR analysis (Supplementary Table S1). The analysis tools used in this study do not allow datasets of mixed ploidy level. Calculations of statistical parameters for the diploid dataset of 1085 cultivars were performed with the Cervus v3.0.7 software using default settings [45,46]. These parameters include number of average alleles *N_a_*, observed heterozygosity *H_obs_*, expected heterozygosity *H_exp_,* polymorphism information content (PIC), average non-exclusion probability of identity of two unrelated individuals (PID) and average non-exclusion probability of identity of two siblings (PIDsib). The number of effective alleles *N_e_* was calculated as follows:

(1) $$N_{e}=1/1-H_{exp}$$

where *H_exp_* is the expected heterozygosity per locus [47,48]. This calculation and the plot showing the probability of identity were performed in Microsoft Excel 2016 and PowerPoint 2016 [49,50]. A genotype curve was compiled with the poppr package v2.9.5 with R v4.4.0 software in RStudio v2024.4.1.748 [51,52,53,54].

### 5.3. STRUCTURE Analysis and Phylogenetic Tree

A dataset of 569 diploid apple cultivars with information on their age and origin was compiled to examine their genetic structure with STRUCTURE v2.3.4 software [55,56] (Supplementary Table S1). Initial statistics for the pairwise population matrix of Nei genetic identity between age groups and the number of average alleles *N_a_* per age group were calculated with GenAlex v6.5 [57]. The STRUCTURE program was run for K = 4 with a 1,000,000 burn-in period and 1,000,000 MCMC steps. K = 4 was chosen in accordance with the four age groups where cultivars were classified based on the year of emergence or breeding (Table 7). The algorithm was run with the LOCPRIOR model using this age classification of the specific cultivars. Information on age was grouped according to Table 7 (Supplementary Table S2). For visualization purposes, the cultivars were also grouped by origin, but this information was not used in the STRUCTURE model (Table 7, Supplementary Table S2). Raw data of the STRUCTURE analysis were exported and compiled to the presented figures with the help of R v4.4.0, RStudio v2024.4.1.748, Microsoft Excel 2016 and PowerPoint 2016 [49,50,53,54]. Next to base R, the packages openxlsx v4.2.5.2, dplyr v1.1.4., ggplot2 v3.5.1. and tidyr v1.3.1. were used [58,59,60,61]. The phylogenetic tree was constructed with DARwin v6.0 software using the unweighted neighbor-joining method [62]. Missing data were dealt with by removing loci with missing data, while 95% of valid data were required for each unit pair. The phylogenetic tree was drawn in the web-based tool Interactive Tree of Life (iTOL) v6 [63]. Within the tool, the labels were manually colored to represent the corresponding age groups, and final editing was performed in Microsoft PowerPoint 2016 [50].

### 5.4. Parentage Analysis

A parentage analysis was performed to infer the direct relationships between 253 offspring cultivars and their respective parent(s). These 253 out of 1085 diploid genotypes had parentage information available from various sources in the literature [23]. The offspring and the respective parent(s) amounted to 315 cultivars in total. For 128 cultivars, both parents were suggested, and for 100 and 25, the maternal and paternal parent was given, respectively. In Cervus v3.0.7 software, a simulation for a parent pair with known sexes was run based on the allele frequency data of the diploid dataset of 1085 cultivars [45,46]. The simulation settings were adjusted for 100,000 offspring, 88 candidate mothers with 0.9 proportion sampled, 69 candidate fathers with 0.9 proportion sampled, 0.997 proportion of typed loci, 0.01 proportion of mistyped loci and 15 minimum typed loci. Delta confidence levels were calculated with default settings. Lastly, parentage analysis was performed for a parent pair of known sexes to infer the most likely parent pair.

## Figures and Tables

**Table 1:** Genetic parameters of 17 SSR markers used in the molecular assessment of 1085 diploid apple cultivars of the GFG.

Locus	Assessed Genotypes	*N_a_* *	*N_e_* *	*H_obs_* * (Locus)	*H_exp_* * (Locus)	PIC *	PID *	PIDsib *
Ch01h01	1085	22	7.04	0.86	0.86	0.84	0.04	0.33
Ch01f07a	1085	18	7.30	0.87	0.86	0.85	0.03	0.33
CH03d07	1083	26	7.35	0.88	0.86	0.85	0.03	0.33
CH04c07	1085	19	7.46	0.88	0.87	0.85	0.03	0.33
CH05f06	1085	14	6.33	0.84	0.84	0.82	0.04	0.34
CH01f03b	1084	14	4.90	0.81	0.80	0.77	0.07	0.37
GD12	1085	20	3.36	0.70	0.70	0.68	0.11	0.43
CH02d08	1083	20	6.54	0.83	0.85	0.83	0.04	0.34
CH05e03	1073	38	9.01	0.65	0.89	0.88	0.02	0.31
CH02c09	1083	17	6.62	0.86	0.85	0.83	0.04	0.34
CH02g09	1085	26	9.01	0.89	0.89	0.88	0.02	0.31
CH01f02	1085	27	8.55	0.88	0.88	0.87	0.03	0.32
CH04f10	1050	37	8.00	0.56	0.88	0.87	0.03	0.32
COL	1083	17	5.08	0.78	0.80	0.78	0.07	0.37
Hi02c07	1085	18	5.10	0.80	0.80	0.78	0.06	0.36
CH04e05	1085	26	4.18	0.79	0.76	0.74	0.08	0.39
GD147	1085	16	5.68	0.83	0.82	0.80	0.05	0.35
Mean	1081.71	22.06	6.56	0.81	0.84	0.82	0.05	0.34

* *N_a_*: number of different alleles; *N_e_*: number of effective alleles, *H_obs_*: observed heterozygosity; *H_exp_*: expected heterozygosity, PIC: polymorphic information content, PID: average non-exclusion probability of identity of two unrelated individuals, PIDsib: average non-exclusion probability of identity of two siblings.

**Table 2:** Genetic parameters for age groups among 569 apple genotypes. Age groups are named after respective age range of apple cultivars and tested against each other for Nei genetic identity.

	n *	*H_exp_* *	*N_a_*	Pairwise Population Matrix of Nei Genetic Identity			
				before 1849	1850 to 1899	1900 to 1949	1950 to present
before 1849	156	0.83	15.65	1			
1850 to 1899	137	0.84	15.53	0.98	1		
1900 to 1949	139	0.82	13.00	0.962	0.965	1	
1950 to present	137	0.81	12.24	0.894	0.899	0.941	1

* n: number of genotypes per group; *H_exp_*: expected heterozygosity; *N_a_*: average number of alleles.

**Table 3:** Cluster proportion of 569 apple genotypes across age groups in %. Age groups are named after the respective age range of apple cultivars. Clusters are inferred based on STRUCTURE analysis of 17 SSR markers at predefined K = 4.

	C1 (in %)	C2 (in %)	C3 (in %)	C4 (in %)	Total (in %)
before 1849	78.2	14.1	7.1	0.6	100
1850 to 1899	72.3	15.3	11.7	0.7	100
1900 to 1949	46	21.6	23.7	8.6	100
1950 to present	15.3	10.9	29.9	43.8	100

**Table 4:** Significant results of parentage analysis for 110 apple cultivars. Significance value of trio confidence was >95%. Alphabetical order by cultivar name. Full result table can be viewed at Supplementary Table S3.

Cultivar Name	Candidate Mother	Candidate Father
Akane	Jonathan	Worcester Parmäne
Alkmene	Geheimrat Dr. Oldenburg	Cox Orange
Allington Pepping	Cox Orange	Goldparmäne
Angold	Antonovka	Golden Delicious
Apollo	Cox Orange	Geheimrat Dr. Oldenburg
Arlet	Golden Delicious	Idared
Aroma	Ingrid Marie	Filippa
Astillisch	Roter Astrachan	Signe Tillisch
Auralia	Cox Orange	Schöner aus Nordhausen
Bancroft	Forest	McIntosh
Berlon	Goldrenette Freiherr von Berlepsch	Ontario
Charles Roß	Cox Orange	Peasgoods Sondergleichen
Charlotte	McIntosh	Greensleeves
Clivia	Geheimrat Dr. Oldenburg	Cox Orange
Cripps Pink	Lady Williams	Golden Delicious
Cripps Red	Lady Williams	Golden Delicious
Delcorf	Jongrimes	Golden Delicious
Delikates	James Grieve	Cortland
Delorina	Blushing Golden	Florina
Discovery	Worcester Parmäne	Schöner aus Bath
Early McIntosh	Weißer Klarapfel	McIntosh
Ecolette	Elstar	Coop 2
Elan	Golden Delicious	James Grieve
Elektra	Cox Orange	Geheimrat Dr. Oldenburg
Elstar	Golden Delicious	Ingrid Marie
Empire	McIntosh	Delicious
Ernst Bosch	Manks Küchenapfel	Ananasrenette
Fantazja	McIntosh	Linda
Fiesta	Cox Orange	Idared
Freyberg	Cox Orange	Golden Delicious
Früher Victoriaapfel	Lord Grosvenor	Keswick’s Küchenapfel
Frureru	Red Winter	Rafzubin
Gala	Kidd’s Orange Red	Golden Delicious
Geheimrat Dr. Oldenburg	Ananasrenette	Kaiser Alexander
Gloria	Gloster	James Grieve
Gloster	Weißer Winterglockenapfel	Delicious
Goldjon	Golden Delicious	Jonathan
Greensleeves	Golden Delicious	James Grieve
Havelgold	Undine	Clivia
Holiday	Macoun	Jonathan
Idagold	Wagenerapfel	Esopus Spitzenburg
Idared	Jonathan	Wagenerapfel
Iduna	Golden Delicious	Weißer Winterglockenapfel
Ingol	Ingrid Marie	Golden Delicious
Ivette	Cox Orange	Golden Delicious
Jamba	Melba	James Grieve
Jan Steen	Rote Sternrenette	Cox Orange
Jester	Worcester Parmäne	Golden Delicious
Jonadel	Jonathan	Delicious
Jonamac	McIntosh	Jonathan
Julia	Quinte	Discovery
Katja	James Grieve	Worcester Parmäne
Kidd’s Orange Red	Cox Orange	Delicious
Laxtons Triumph	Goldparmäne	Cox Orange
Lonjon	London Pepping	Jonathan
Lord Lambourne	James Grieve	Worcester Parmäne
Maigold	Fraurotacher	Golden Delicious
Margol	Ingrid Marie	Golden Delicious
Marina	Kidd’s Orange Red	Idared
Melrose	Jonathan	Delicious
Merton Beauty	Ellisons Orangenpepping	Cox Orange
Merton Charm	McIntosh	Cox Orange
Millicent Barnes	Gascoynes Scharlachroter	Cox Orange
Milton	Weißer Klarapfel	McIntosh
Monarch	Peasgoods Sondergleichen	Dumelows Seedling
Monrö	Jonathan	Rome Beauty
Neujahrsapfel	London Pepping	McIntosh
Odin	Golden Delicious	Ingrid Marie
Ontario	Wagenerapfel	Northern Spy
Orangenburg	Cox Orange	Geheimrat Dr. Oldenburg
Orleans Renette	Karmeliter Renette	Französische Edelrenette
Pia	Idared	Helios
Pidi	Britemac	Coop 2
Piflora	Idared	Golden Delicious
Pikant	Undine	Carola
Pikkolo	Clivia	Auralia
Pikosa	Pirella	Idared
Pilana	Pirella	Idared
Pilot	Clivia	Undine
Pimona	Clivia	Auralia
Pinett	Idared	Bancroft
Pingo	Idared	Bancroft
Pinova	Clivia	Golden Delicious
Pirella	Golden Delicious	Alkmene
Pirina	Alkmene	Geheimrat Dr. Oldenburg
Piros	Helios	Apollo
Pivita	Pinova	Idared
Pohorka	Cox Orange	Ontario
Prinzeßin Irene	Jonathan	Cox Orange
Quinte	Crimson Beauty	Melba
Rafzubin	Golden Delicious	Cox Orange
Rebella	Golden Delicious	Remo
Rekarda	Golden Delicious	Remo
Rival	Peasgoods Sondergleichen	Cox Orange
Rubin	Golden Delicious	Lord Lambourne
Rubinola	Coop 2	Rubin
Sansa	Gala	Akane
Schöner aus Mlejew	Goldparmäne	McIntosh
Schweizer Orangenapfel	Ontario	Cox Orange
Shampion	Golden Delicious	Lord Lambourne
Shinsei	Golden Delicious	Early McIntosh
Slava Pobeditelaam	Weißer Klarapfel	McIntosh
Sommerregent	Anton Fischer	James Grieve
Spencer	McIntosh	Golden Delicious
Summerland	McIntosh	Golden Delicious
Telamon	McIntosh	Golden Delicious
Tuscan	McIntosh	Greensleeves
Tydemans Early	McIntosh	Worcester Parmäne
Tydemans Oktoberpepping	Cox Orange	Ellisons Orangenpepping
Weißer Winterglockenapfel	Boikenapfel	Prinzenapfel

**Table 5:** Summary of the parentage analysis for 253 cultivars.

	n	Both Parents Confirmed	Mother Confirmed/Father Rejected	Father Confirmed/Mother Rejected	Both Parents Rejected
Both parents suggested	128	110	12	3	3
Mother suggested	100	-	82	18	-
Father suggested	25	-	7	18	-

**Table 6:** Overview of the trueness-to-type criteria used by the GFG for evaluating the authenticity of cultivar identification.

German Fruit Genebank Trueness-to-Type Criteria		Explanation
0	deceased	tree died during project duration
1	true-to-type	pomologically and molecularly assessed
2	true-to-type (group/mutant)	pomologically and molecularly assessed
3	not determined	no references available, cultivar name unknown
4	not assessed	not assessed (pomologically)
		pomologically assessed, not assessed molecularly
5	true-to-type with reservations	molecularly assessed for at least 3 accessions and pomologically assessed for at least 2 accessions
		pomologically assessed with reservation, molecularly assessed

**Table 7:** Categories for age and origin of apple genotypes used in the analysis of genetic structure. Age and origin group names refer to the respective age range or geographic origin of apple cultivars.

Category	Group Name	n *
Age	Before 1849	156
	1850 to 1899	137
	1900 to 1949	139
	1950 to present	137
Origin	Germany	264
	Europe	208
	Other	97

* n = number of apple genotypes per group.

## Data Availability

This study used an open access dataset that can be found in the following online repository: [13].

## Supplementary Materials

The following supporting information can be downloaded at https://www.mdpi.com/article/10.3390/plants13192699/s1, Figure S1: Genotype accumulation curve, Figure S2: Genetic structure analysis in the context of apple cultivar origins, Figure S3: Phylogenetic tree of 569 apple cultivars, Table S1: Overview of data annotation and usage, Table S2: Compiled STRUCTURE output, Table S3: compiled cervus (parentage analysis) output, Textfile S1: Raw STRUCTURE output and metadata.
