# Supplementary material for: Pomological and Molecular Characterization of Apple Cultivars in the German Fruit Genebank
Source: Plants (Basel). 2024 Sep 26;13(19):2699. doi: 10.3390/plants13192699 (PMC11478905; doi:10.3390/plants13192699)
Supplement: Supplementary file 1 [file plants-13-02699-s001.zip › MDPI_Supplementary_Figure_S1_S2_S3_descriptions.pdf]

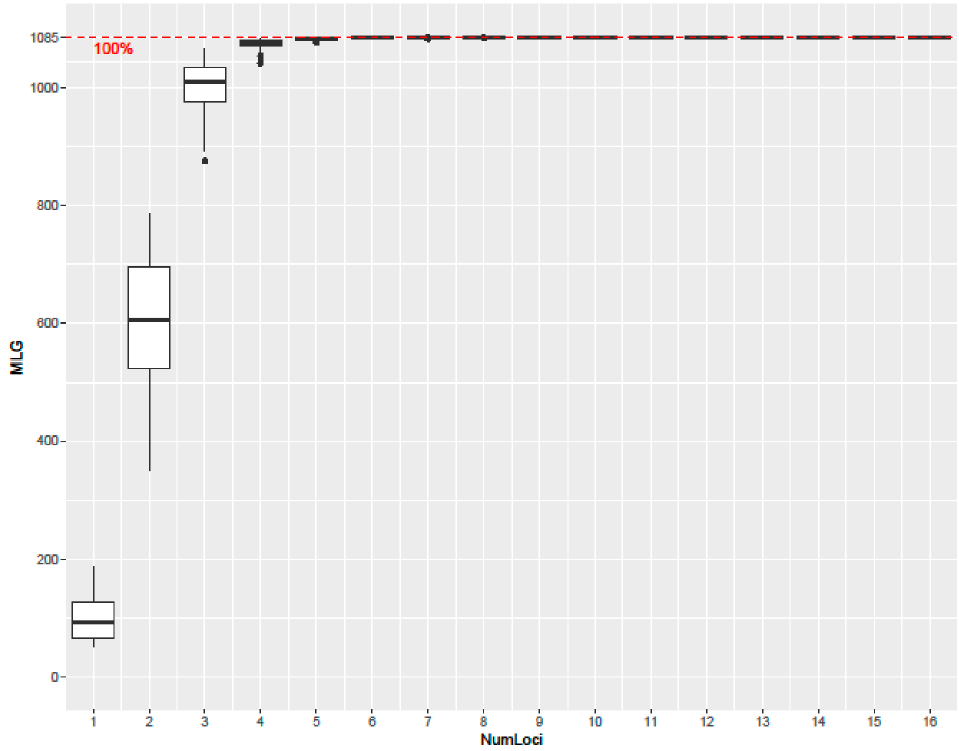

Supplementary Figure S1: Genotype accumulation curve *of 17 SSR markers and 1,085 diploid apple genotypes constructed by R package poppr. The boxplots show how many markers (NumLoci) are needed to distinguish the respective number of multilocus genotypes (MLG). To distinguish the 1,085 diploid genotypes with near 100% certainty, at least 6 markers are needed.*

MDPI_Supplementary_Figure_S2.svg (opens in browser)

Supplementary Figure S2: Phylogenetic tree of the genetic structure analysis results *in high resolution. Cultivars are marked according to their respective dominant cluster (1 to 4) based on the inferred membership fraction. The clusters are generated by STRUCTURE software with K = 4. Per cluster, the cultivar with the highest inferred membership fraction is marked with red font colour.*

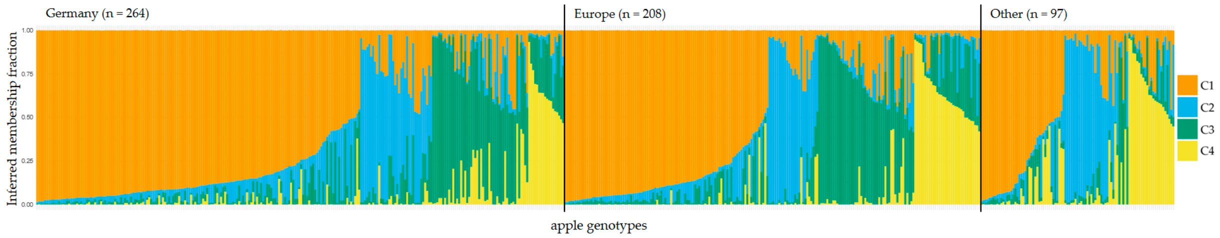

Supplementary Figure S1: Genetic structure among 569 apple genotypes *based on 17 SSR markers with predefined K = 4. Each genotype is represented by a vertical bar that is partitioned in colour according to the inferred membership fraction to clusters 1 to 4 (C1 to C4). The data is sorted by Q-value per origin group.*
