# Supplementary figures and images for: Pomological and Molecular Characterization of Apple Cultivars in the German Fruit Genebank
Source: Plants (Basel). 2024 Sep 26;13(19):2699. doi: 10.3390/plants13192699 (PMC11478905; doi:10.3390/plants13192699)

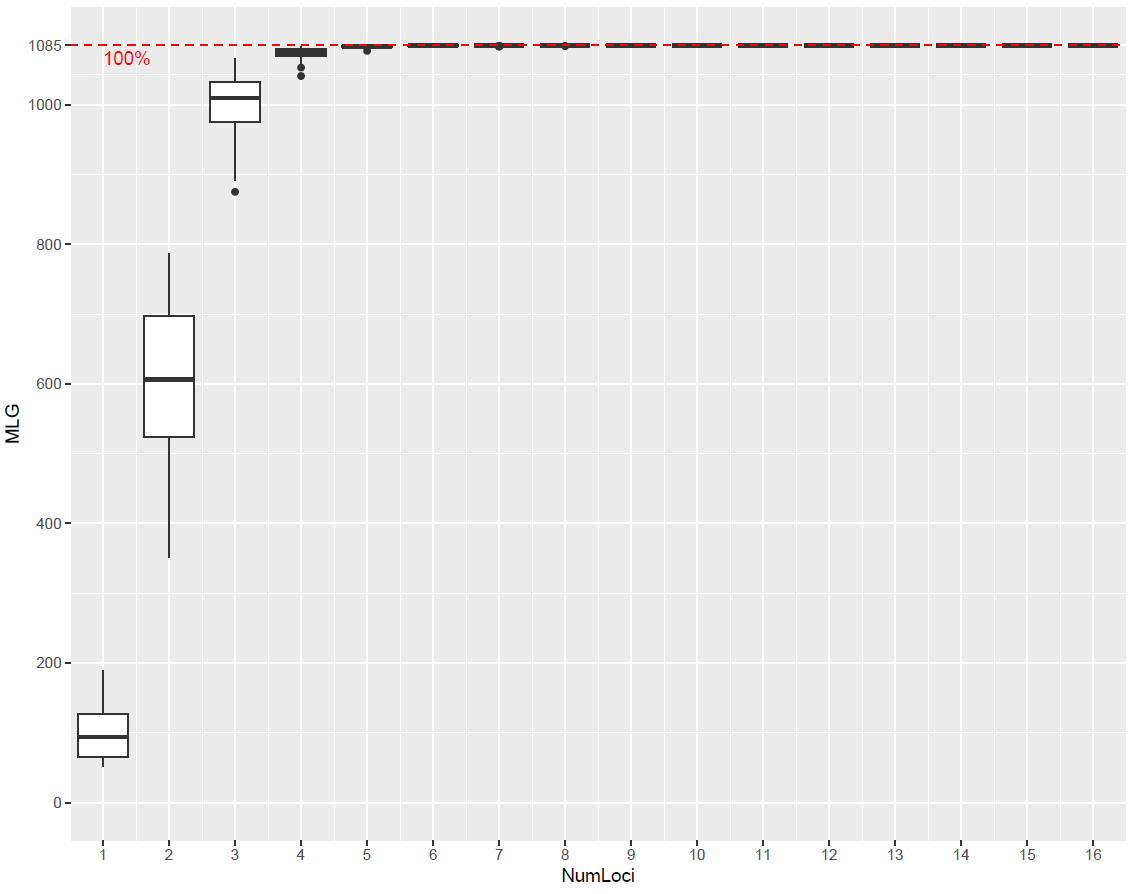

Supplement: Supplementary file 1 [file plants-13-02699-s001.zip › MDPI_Supplementary_Figure_S1.png]

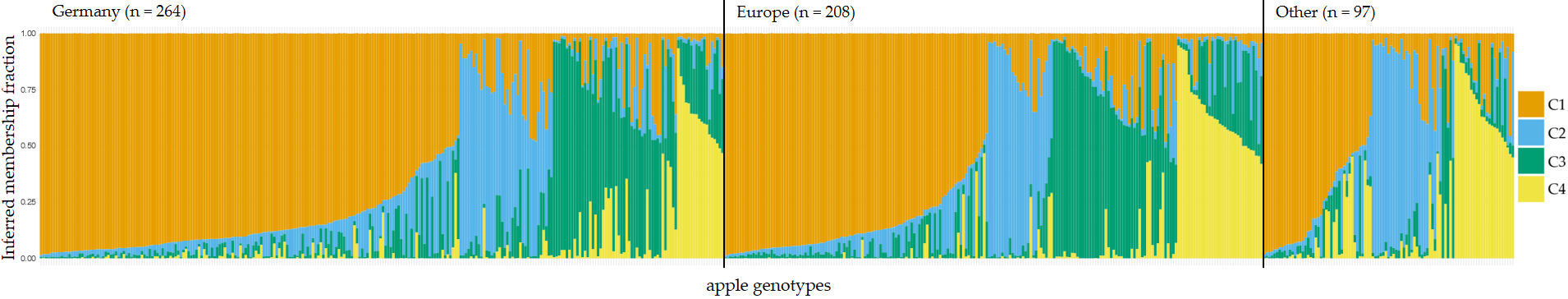

Supplement: Supplementary file 1 [file plants-13-02699-s001.zip › MDPI_Supplementary_Figure_S3.png]
